# Structures of ABC transporters: handle with care

**DOI:** 10.1002/1873-3468.13966

**Published:** 2020-11-21

**Authors:** Oded Lewinson, Cédric Orelle, Markus A. Seeger

**Affiliations:** ^1^ Department of Molecular Microbiology and the Rappaport Institute for Medical Sciences Faculty of Medicine The Technion‐Israel Institute of Technology Haifa Israel; ^2^ CNRS Molecular Microbiology and Structural Biochemistry (MMSB, UMR 5086) University of Lyon Lyon France; ^3^ Institute of Medical Microbiology University of Zurich Zurich Switzerland

**Keywords:** ABC transporters, conformational changes, energy landscape, membrane proteins, single molecule, structural biology, structure function, transport mechanism

## Abstract

In the past two decades, the ATP‐binding cassette (ABC) transporters' field has undergone a structural revolution. The importance of structural biology to the development of the field of ABC transporters cannot be overstated, as the ensemble of structures not only revealed the architecture of ABC transporters but also shaped our mechanistic view of these remarkable molecular machines. Nevertheless, we advocate that the mechanistic interpretation of the structures is not trivial and should be carried out with prudence. Herein, we bring several examples of structures of ABC transporters that merit re‐interpretation *via* careful comparison to experimental data. We propose that it is of the upmost importance to place new structures within the context of the available experimental data.

## Graphical abstract

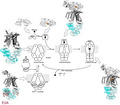

## Abbreviations

**cryo‐EM**, cryogenic electron microscopy

**EPR**, electron paramagnetic resonance

**NBDs**, nucleotide‐binding domains

**SBP**, substrate‐binding protein

**smFRET**, single‐molecule fluorescence resonance energy transfer

**TMDs**, transmembrane domains

ATP‐binding cassette (ABC) proteins constitute one of the largest and ancient protein superfamilies and are found in all living organisms [1, 2]. These proteins utilize the energy from ATP binding and hydrolysis to perform diverse mechanical functions. Most ABC proteins are membrane proteins transporting a variety of molecules across cellular membranes, including ions, vitamins, amino acids, peptides, proteins, polysaccharides, drugs, lipids, or cholesterol. An ABC transporter is minimally comprised of two transmembrane domains (TMDs) and two cytoplasmic nucleotide‐binding domains (NBDs). While the sequence of NBDs is highly conserved, the TMDs are highly variable from one transporter to another depending on the molecule(s) translocated.

## Crystal structures of ABC transporters: a bumpy start followed by a golden age

In 2001, Chang and Roth published the first crystal structure of an ABC transporter, the bacterial lipid A exporter MsbA [3]. Five years later, however, Dawson and Locher published the structure of another exporter, Sav1866, that cast serious doubts on MsbA structure [4]. Further investigations revealed that the structure determination of MsbA was carelessly performed [5], and this blunder led to dramatically erroneous structures of not only MsbA but also EmrE from the SMR family [6]. The subsequent retraction of five high‐profile publications provided a forceful warning of the harmful consequences of using low‐quality electron density data.

Fortunately, the pioneering work by Locher and Rees quickly followed and the landmark first crystal structure of an ABC transporter (the *Escherichia coli* vitamin B_12_ importer BtuCD) was published in 2002 [7]. This began a 20‐year exciting era of structural determination of ABC transporters, which was nourished by the functional and structural diversity of the superfamily. Not only did the structural information shape our mechanistic view of this fascinating family of proteins, it also provided a basis for focused functional analysis [8, 9], facilitated the introduction of fluorescent and spin labels [10, 11, 12, 13, 14, 15, 16], and enabled unprecedented molecular simulations studies [9, 17]. As a result, the interest in ABC proteins has been continuously rising and their structural–functional classification is still evolving [1, 18, 19].

## The mechanistic interpretation of structures

The extensive number of available structures widened our view of the architecture of ABC transporters and drove us away from a strictly unified transport mechanism. Although the importance of structures to the development of the ABC field cannot be overemphasized, we stress that the mechanistic interpretation of these structures is sometimes not trivial and should be rigorously carried out in light of experimental data. In this review, we present several examples of crystal structures of ABC transporters that merit re‐interpretation on the basis of biochemical studies. These transporters include the two best characterized importers in the field, for example, BtuCD and MalFGK_2_, while examples of ECF‐type ABC transporters were recently discussed elsewhere [20]. Finally, we will analyze the case of exporters since many fundamental questions remains unanswered despite the availability of extensive structural data.

## The *E. coli* vitamin B_12_ transporter BtuCD: A missing link and an off‐trajectory conformation

The first structure of the *E. coli* vitamin B_12_ ABC transporter BtuCD, published in 2002 by Locher *et al*. [7] marked the beginning of the golden era of structure determination of ABC transporters. Subsequent innovative work by Locher *et al*. provided snapshots of BtuCD in its pretranslocation, high‐energy intermediate, and post‐translocation states [21, 22, 23] and established BtuCD as the archetypical Type II ABC importer. These structures of high technical quality provided an invaluable framework for biochemical, biophysical, and mutagenesis studies and today BtuCD is often used as a benchmark for structure–functions studies.

The crystal structure of apo BtuCD revealed that in its nucleotide‐free state BtuCD adopts an outward‐facing (OF) conformation, with the NBDs slightly separated (~ 14 Å between the P‐loop of one monomer to the ABC signature motifs of the other). Upon binding of ATP (mimicked by AMP‐PNP,[23]), major structural rearrangements are observed, comprising both the TMDs and NBDs. The NBDs come closer together and the cytosolic end of the pore‐lining TM helices swing out to yield a conformation that is now more open toward the cytoplasm. The volume of the translocation channel increases, making more room to accommodate vitamin B_12_. Docking of BtuF to nucleotide‐bound BtuCD mostly leads to structural rearrangements of extracellular side of the TMDs helices, which form the site where the substrate‐binding protein (SBP) docks. The nucleotide‐free BtuF‐bound conformation [21] is suggested to represent a post‐translocation state, an intermediate step toward the return to the apo ground state.

As already mentioned, these structures provided a wealth of information and many subsequent studies would not have been otherwise possible. However, we would like to argue that the mechanistic interpretation of these structures is not straightforward and should be approached with caution.

## The elusive inward‐facing conformation

Others and we have used the four available crystal structures of BtuCD to describe the transport cycle of BtuCD [9, 24, 25, 26]. However, what is clearly missing is the structure of the inward‐facing (IF) conformation, as observed for example for the ABC transporters (importers) for methionine (MetNI [27]) and tungstate/molybdate transporter (MolBC, [28]).

In all four available BtuCD structures, the cytosolic side of the TMDs is too narrow to allow passage of vitamin B_12,_ leading to the inevitable conclusion that the current structures do not cover the complete amplitude of the structural changes that are necessary for the transmembrane translocation of vitamin B_12_. Therefore, attempts to describe the complete transport cycle of BtuCD using only the available structures are bound to be incomplete. Possibly, the IF conformation is disfavored under crystallization conditions and is therefore difficult to trap. Alternatively, the IF conformation of BtuCD may be short lived. This latter possibility is supported by single‐molecule FRET (smFRET) studies of BtuCD. In these studies [24, 25], we could detect conformational fluctuations that correspond to the opening and closing of the NBDs and movements of the TMDs. However, the smFRET changes measured at the cytoplasmic side of the TMDs were small and unlikely correspond to a transition to the fully inward‐open state. Possibly, the dwell time in the elusive fully inward‐open state is very short, shorter than the nominal 50 ms temporal resolution of these smFRET measurements.

In the same work, we used smFRET to compare the conformational changes that take place under the crystallization conditions (detergent solution) or in a membrane‐mimetic environment (nanodiscs, [24]). We observed that in the membrane, the opening of OF apo BtuCD toward the periplasm is wider than the one observed in the corresponding crystal structure [7]. Moreover, in the membrane, docking of BtuF leads to a more pronounced transformation to the occluded state [24], manifested by a tighter seal of the periplasmic gate.

Together, these observations imply that in the membrane, the amplitude of BtuCD's conformational changes and its conformational space is more expansive than what is covered by the crystal structures.

## An off‐trajectory conformation trapped by cross‐linking?

Additional uncertainty surrounds the interplay between ATP and BtuF binding by BtuCD. Structural alignment of the BtuF‐ and BtuF/AMP‐PNP‐bound structures reveals an RMS of 2.65 Å (for 1080 Cα), which is mostly localized to the NBDs and to the cytoplasmic side of the TMDs. In comparison, residues that comprise the docking site for BtuF [21] are effected to a much lesser degree by binding of ATP. At face value, the relative insensitivity of the BtuF docking sites to binding of ATP suggests that the affinity of the BtuCD‐BtuF interaction is insensitive to binding of ATP. However, several independent lines of evidence do not support this interpretation.

Using surface plasmon resonance (SPR), we observed that binding of ATP reduces the BtuCD‐BtuF interaction affinity by ~ 100‐fold, and comparable results were obtained in interactions studies conducted with reconstituted liposomes [29]. Similar conclusions were reached by Fiorentino *et al*. [26] who used native mass spectrometry and found that binding of ATP by BtuCD destabilizes the BtuCD‐F complex.

**Fig. 1:**
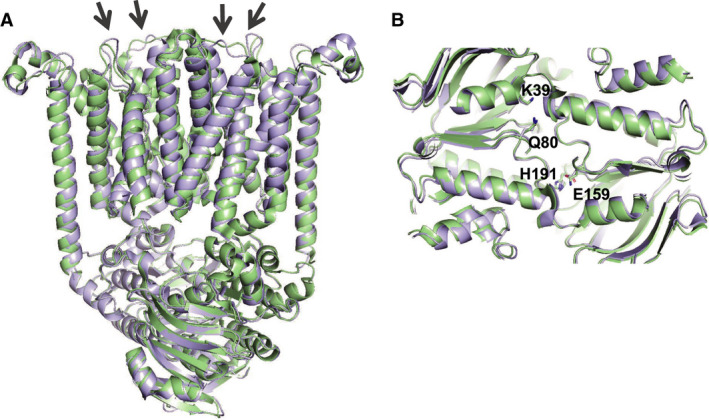
Effects of BtuF docking on the conformation of BtuCD. Shown is a cartoon representation of the structural alignment of BtuCD‐AMP‐PNP (PDB 4R9U, cyan) and BtuCD‐F‐AMP‐PNP (PDB 2QI9, green). BtuF was omitted for clarity (A) Side view from along the membrane plane, arrows indicate TMD loops that interact with BtuF. (B) Top view of the NBD interface, highlighting their overall unchanged conformations and positions of the conserved ATP binding residues K39 (Walker A), Q80 (Q loop), E159 (Walker B), and H191 (Switch region).

The thermodynamic flip coin of the ATP‐BtuF relationship is that if ATP lowers the BtuF:BtuCD binding affinity, BtuF must have the same effect of binding of ATP by BtuCD. However, this is not evident in the crystal structures: A comparison of the AMP‐PNP‐ and AMP‐PNP/BtuF‐bound crystal structures reveals that docking of BtuF almost exclusively affects TMD residues that comprise its own docking site (Fig. 1A). In contrast, the conformations of the NBDs remain practically identical (RMS of 0.36 for 472 C α) with identical positions for the ATP‐ligating residues (Fig. 1B). Again, a simplistic interpretation of the structures (i.e. ignoring that the nucleotide concentration used to solve the two structures was possibly way above its *K*_D_) would imply that the BtuCD:ATP interaction is unaffected by BtuF. Indeed, such a conclusion contrasts with experimental evidence. For example, in smFRET experiments, we observed that the tendency of the NBDs to open is greater once BtuF is docked [24]. Since the ATP‐binding sites are located at the NBDs dimer interface, opening of the NBDs disrupts nucleotide binding and lowers the ATP‐binding affinity [23]. Importantly, this NBDs‐opening effect of BtuF was only observed in a membrane‐mimetic environment, and not in detergent solution. This may explain why a BtuF‐induced opening of the NBDs was not captured in the crystal structures, which were obtained in detergent solution.

Given the above considerations and experimental data we suggest that binding of BtuF and ATP by BtuCD are allosterically negatively correlated, in the sense that binding of ATP drives the dissociating of BtuF, and *vice versa*. Contradictory to this suggestion, the tripartite complex was successfully crystallized and the structure of BtuCD‐AMP‐PNP‐BtuF was solved [22]. An often‐ignored detail of this structure is that crystal formation required chemical cross‐linking of the NBDs, which by definition leads to a shift of the conformational equilibriums toward closed NBDs with bound nucleotides. Without the cross‐link, attempts to crystallize BtuCD–F with bound ATP always yielded the nucleotide‐free form, even when the Walker B (E159Q) mutant was used in conjunction with the nonhydrolysable ATP analogue AMP‐PNP [22]. In our view, cross‐linking of the NBDs prevented their normal response to docking of BtuF, which is to open. We therefore suggest that the BtuCD‐AMP‐PNP‐BtuF structure may represent an off‐trajectory conformation, or one which is sparsely populated.

## Crystal structures of MalEFGK_2_: shaping the view of the transport cycle

### The alternating access mechanism

**Fig. 2:**
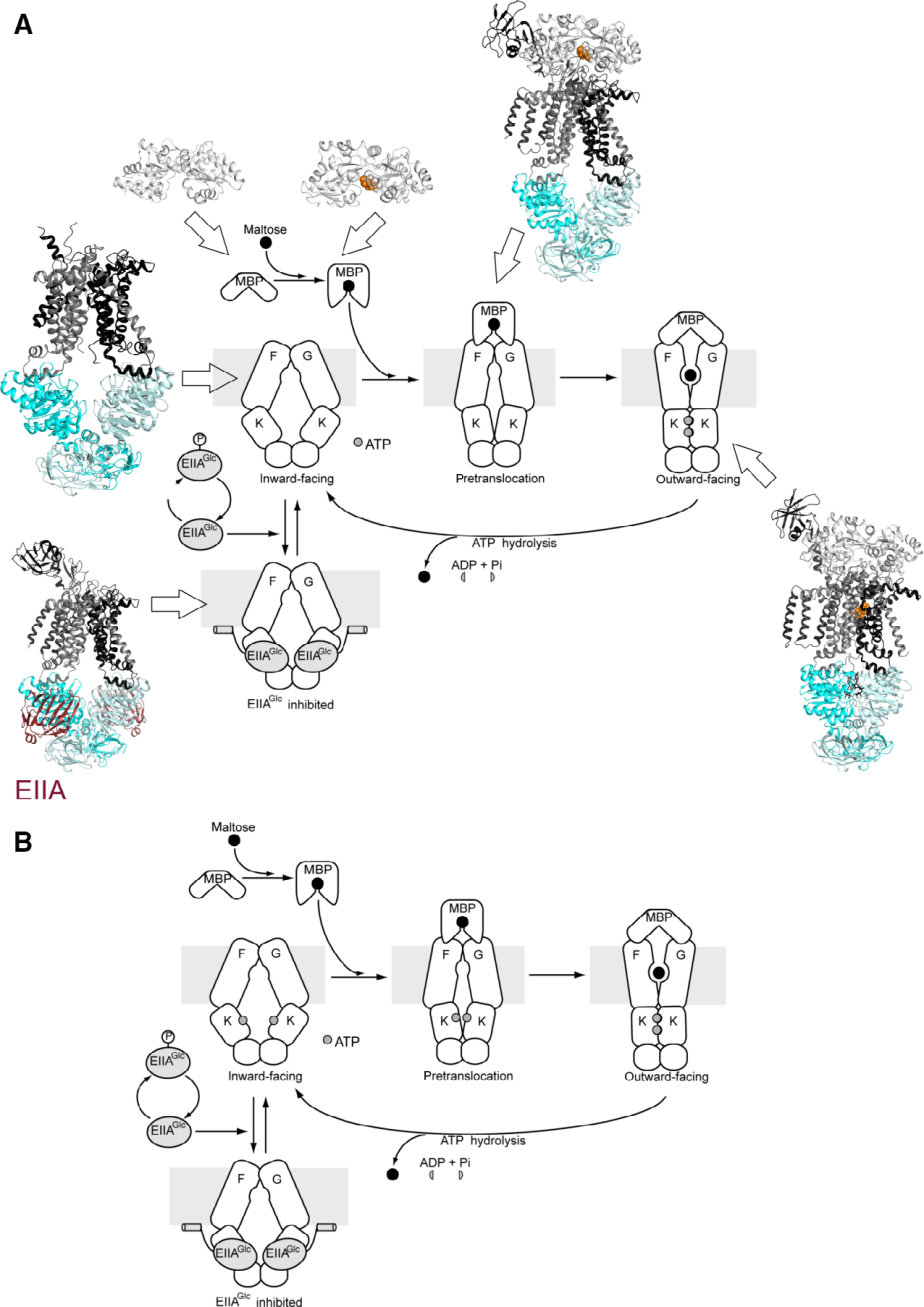
Transport cycle of the maltose ABC transporter. (A) Various crystal structures of the maltose import system in *E. coli* allowed to hypothesize the transport scheme as indicated (IF: PDB 3FH6; pretranslocation: PDB 3PV0; OF: PDB 2R6G; EIIA^Glc^ inhibited: PDB 4JBW). In this model, interaction with maltose‐bound MBP in the periplasm induces a partial closure of the MalK dimer in the cytoplasm. ATP binding to this conformation then promotes progression to the OF state. The EIIA protein is involved in catabolite repression and was shown to stabilize the transporter in an IF conformation and to prevent the structural rearrangements necessary for ATP hydrolysis [101]. Figure adapted from [34, 101]. (B) EPR experiments from Davidson's and Bordignon's laboratories suggest a different transport cycle in which ATP binding contributes to the stabilization of the pretranslocation state. Note that in both A and B, the MBP promotes essential conformational changes in the NBDs .

The maltose ABC transporter from *E. coli* is composed of two TMDs, MalF and MalG, and a homodimer of NBDs, MalK_2_. The periplasmic maltose‐binding protein (MBP or MalE) delivers maltose and other maltodextrins to the transporter. Both apo and holo MalE stimulate the ATPase activity of the transporter, with the latter ~ 7–10‐fold more efficiently so [30]. Crystallographic work performed on this type I import system (MalEFGK_2_) provided detailed molecular insights into the transport mechanism [31]. The IF state of the transporter was crystallized in the absence of nucleotides and MalE, and was interpreted as being the resting state [32]. In this conformation, the NBDs were well separated and the TMDs form an IF cavity that exposes the transmembrane maltose‐binding site to the cytoplasm. The structure of the OF state was achieved by cocrystallizing a catalytic mutant (E159Q) of the transporter with ATP, maltose, and MalE [33]. In this state, maltose is occluded within a solvent‐filled cavity in the upper half of the transmembrane subunits. The binding protein docks onto the entrance of the cavity in an open conformation and shields the cavity. Based on these structures, the import of maltose was suggested to proceed by an alternating access mechanism in which the TMDs switch between inward‐ and OF conformations, alternately exposing the transmembrane maltose‐binding site to opposite sides of the membrane. These conformational changes in the TMD are driven by ATP binding and hydrolysis events occurring in MalK_2_. These two crystal structures of MalEFGK_2_ were largely consistent with functional and biochemical studies (see for recent review [34]) and were compatible with the formerly proposed ‘concerted’ model of transport. In this model, the concomitant closure of the MalK dimer and opening of MalE release maltose to the transmembrane cavity [30] (Fig. 2A).

### Bringing to light the intermediate states

Additional work was necessary to elucidate the roles of ATP binding and MalE docking in the transport mechanism. Since there was no crystal structure of ATP‐bound MalFGK_2_ (i.e. in the absence of MalE), the effect of ATP binding to the transporter was investigated by site‐directed spin labeling and electron paramagnetic resonance (EPR) spectroscopy. Distance measurements were used to track the opening and closing events of MalK_2_ during the catalytic cycle of the transporter [12].

Depending on the ligands, MalK_2_ was observed in three different conformations: open, closed, and intermediate, the latter being typically called either semi‐open or semi‐closed. In the absence of ligand, MalK_2_ remained in an open conformation, as seen in the resting state structure of MalFGK_2_. Binding of ATP alone did not induce MalK closure in the intact transporter; however, the addition of both ATP and maltose‐bound MalE stabilized a closed MalK dimer. These results strongly supported the idea that maltose‐bound MalE stimulates the ATPase activity of the transporter by promoting MalK closure [12]. Consistent with this, a recent allosteric study suggested that MalE controls the conformational changes in MalEFGK_2_ such that the dynamics of the MalE‐docking sites precedes, and that of the ATP sites follow [9]. Finally, EPR spectroscopy suggested that MalK was semi‐open in the ADP‐bound post‐translocation state [12, 35, 36].

### How to interpret the crystal structures of the pretranslocation state?

The crystal structure of another MalFGK_2_ intermediate, referred to as the pretranslocation state, was solved in the presence of maltose‐bound MalE. Docking of both lobes of maltose‐bound MalE facilitated partial rotations of both the TMDs and the MalK dimer, bringing some critical catalytic residues to the NBD dimer interface [37]. Consequently, the IF cavity formed by the TMDs was narrower, and the MalK dimer was in an intermediate semi‐closed conformation. It was hypothesized that this partial closure of the NBDs would facilitate ATP binding, which would subsequently perturb the pretranslocation state and initiate the transition to the OF conformation (Fig. 2A).

However in numerous EPR experiments from different laboratories, it was repeatedly shown that the addition of liganded MalE *per se* to the transporter does not induce the semi‐closed conformation of the MalK dimer; rather, the NBDs remain in an open conformation [12, 13, 14, 35, 36]. The respective docking of each of the two lobes of MalE was shown to be dependent on the catalytic cycle of the transporter [35, 36]. However, full dissociation of MalE is not a required step for substrate translocation since supercomplexes with MalE either cross‐linked to MalG or fused to MalF are transport‐competent [36, 38].

The pretranslocation state with a semi‐closed MalK dimer was seen in EPR only when AMP‐PNP‐Mg^2+^ was bound and MalE was prevented from opening with an engineered disulfide bond [14]. When wild type MalE was used, the addition of AMP‐PNP‐Mg^2+^ was shown to drive the transporter to the OF state. The hope was that if MalE cannot open, an intermediate state would be revealed. These results suggest that the presence of the nucleotide stabilizes the transition to the MalK semi‐closed state [14]. Importantly, the pretranslocation state was also crystallized with the locked (cross‐linked) version of MalE after soaking the crystal with AMP‐PNP‐Mg^2+^, which was thus in agreement with EPR [37]. Considering the *in vivo* mm concentrations of ATP [39], the transporter is expected to bind ATP relatively frequently and be primed for a cycle of ATP hydrolysis once MalE binds maltose.

By analogy with enzymes, Shilton proposed that conformational changes in the maltose transporter are likely promoted by the stabilization of higher‐energy intermediate conformations [40]. He speculated that the pretranslocation state of MalEFGK_2_ would have a relatively high energy as compared to the inward and OF conformations, thereby presenting an energetic barrier that prevents uncoupled ATP hydrolysis. If maltose‐bound MalE was to bind preferentially to the resting state of MalFGK_2_, it would decrease ATPase activity by stabilizing a low‐energy conformation that is not competent for ATP hydrolysis. Thus, Shilton hypothesized that binding to a higher‐energy intermediate between the inward‐ and OF states offers an energetically reasonable pathway for liganded MalE to promote conformational changes in the system. Maltose‐bound MalE and ATP might synergistically initiate the catalytic cycle by binding to, and lowering the energy of the intermediate conformation, facilitating the transition to the OF conformation (Fig. 2B).

### Properties of the maltose transporter differ in detergent or membrane environments

Last but not least, the dynamics of the maltose transporter was shown to be different in detergent solution or lipids [35]. Since detergents can alter the structural properties of membrane proteins [20], biochemical/biophysical studies are thus essential to analyze and validate the behavior of the transporter in native‐like environments. In the membrane environment, only the substrate‐bound form of MalE efficiently stimulates the ATPase activity. However, when the transporter is in detergent, MalE‐stimulated ATPase activity does not require maltose. Such discrepancies must be kept in mind as all MalFGK_2_ structures published so far were determined in detergent. A recent low‐resolution cryogenic electron microscopy (cryo‐EM) analysis of MalEFGK_2_ was conducted in nanodiscs and could provide a basis for future useful mechanistic investigations [41]. Alternatively, investigation of other detergent alternatives [42, 43] may also be helpful for future structural studies.

## Structural and functional diversity of ABC exporters

### Prevailing and alternative molecular mechanisms of ABC exporters and the role of ATP hydrolysis

ATP‐binding cassette exporters are found in all kingdoms of life. Owing to their paramount role to mediate vital transport processes in the human body, they have been extensively studied for more than 40 years [44]. Human ABC transporters fall into two types of ABC exporter classes [45]. Type I exporters (reannotated as type IV ABC transporters according to the recent classification [46]) are the predominant fold, which all resemble its structural founding member, Sav1866 [4]. Type II exporter folds are found in the human subfamilies ABCA and ABCG (founding member ABCG5/ACGG8 [18]), but are also involved in bacteria in the export of O‐antigens [47]. As the name suggests, ABC exporters are primarily involved in the export of substrates (e.g. drugs, lipids or peptides). However, the type I exporter fold has evolved to fulfill a myriad of additional functionalities and is found in the incarnation of a nucleotide‐gated chloride channel (CFTR) [48], a nucleotide‐sensing regulatory domain of a potassium channel (SUR1) [49, 50], and importer proteins for cobalamin (ABCD4 [51] and Rv1819c [52]) or bacterial siderophores (IrtAB) [53]. Compared with ABC importers, classical ABC exporters are rather simple proteins. In order to pump substrates out of the cell against a chemical gradient, they capture their substrates with moderate affinity (micromolar range) from the cytoplasm or inner membrane leaflet and release them at the other side of the membrane or into the outer membrane leaflet. The accompanying affinity switch (proposed by Higgins, Callaghan, Linton, and coworkers [54, 55]) is accomplished by conformational changes of the TMDs, which alternate between a high affinity binding IF state and a low affinity binding OF state, a process that is energetically coupled to the hydrolysis of ATP by the NBDs.

There are two alternative views regarding the coupling between ATP binding and hydrolysis and conformational changes at the TMDs. According to the first model (which constitutes the prevailing mechanism), binding of two ATP molecules leads to the formation of the closed NBD sandwich dimer and results in a conformational change at the TMDs from the IF to the OF state, which is sufficient to translocate one substrate molecule across the membrane [15, 56, 57, 58, 59]. ATP hydrolysis is then required to initiate the dissociation of the closed NBD dimer, in order to reset the transporter back to its IF state and keep the transport cycle going [60, 61].

However, according to a second model, nucleotide binding is not sufficient to drive the IF‐OF isomerization, but requires ATP hydrolysis. This alternative model is based on the observation that for example P‐glycoprotein (ABCB1) and BmrCD do not switch to the OF state with nonhydrolyzable ATP analogues such as AMP‐PNP, but only if ATP and magnesium (and in case of ABCB1 also vanadate) are present [16, 62].

### ABC exporter apo structures—what are they good for?

**Fig. 3:**
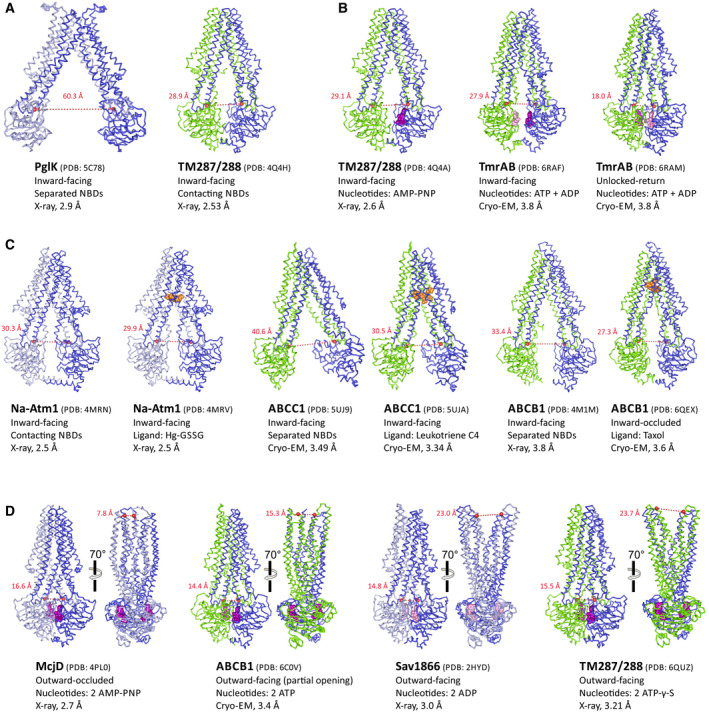
Type I ABC exporter structures at a glance. The transporters are viewed along the membrane plane. Homodimeric ABC exporters are shown in dark and light blue, while heterodimeric ABC exporters are shown in blue and green (regardless whether they are composed of one or two polypeptide chains). Bound nucleotides are depicted as purple (ATP) or pink (ADP) spheres. The distances between the coupling helices—measured between the C_α_‐atoms (highlighted as red sphere) of the residues corresponding to G201 (TM287) and G225 (TM288) of TM287/288—are a measure of NBD opening/closure and are < 17 Å for fully closed NBDs. The distance between the extracellular wings—measured between the C_α_‐atoms of the residues corresponding to D65 (TM288) and T292 (TM288) of TM287/288—are a measure of extracellular gate opening. (A) Two examples of highly resolved apo structures with widely opened (PglK) and contacting (TM287/288) NBDs. (B) IF structures obtained in the presence of AMP‐PNP (bound to the degenerate site of TM287/288) or while hydrolyzing ATP (TmrAB). The unlocked‐return state of TmrAB is an intermediate of the resetting step of the transport cycle and contains ADP in the consensus site resulting in a slight opening of the NBDs. (C) Side‐by‐side comparison of three ABC exporters with and without (apo) bound substrates (orange spheres). Owing to inter‐NBD contacts *via* C‐terminal helices, Na‐Atm1 does change its conformation as a result of substrate binding. In contrast, apo ABCC1 and ABCB1 exhibit fully separated NBDs, which approach as a result of substrate binding. The taxol accommodated by ABCB1 is fully occluded (i.e. inaccessible from the cytoplasm); hence, this state is called inward‐occluded. (D) ABC exporter structures with fully closed NBDs obtained with ATP, ADP, AMP‐PNP, or ATP‐γ‐S, shown from two angles. The structures differ with regard to extracellular gate opening; outward‐occluded (McjD), partial opening (ABCB1), and OF (Sav1866 and TM287/288). Of note, ABCB1 and TM287/288 contained mutations in the Walker B motif to slow down ATP hydrolysis.

A recurrent topic of debate and contemplation are the numerous apo structures of type I ABC exporters, in which the NBDs are far apart and the TMDs assume a tipi tent‐like structure; these structures have been widely considered to be detergent and crystallization artifacts [63, 64, 65] (Fig. 3A). What is highly evident from biochemical and spectroscopic experiments is the fact, that for a majority of type I ABC exporters, the IF apo state is flexible and plastic, while the OF state with its firmly closed NBDs is rigidified [66, 67, 68, 69, 70, 71]. However, a completely nucleotide‐free ABC exporter will rarely exist in the cell, and therefore, the entire discussion pertaining to apo structures and analyses has mostly arisen due to convenience (because apo states can be looked at quite easily *in vitro* by getting rid of the nucleotides during protein purification) and scientific pragmatism (large differences become apparent versus the stiff ATP‐bound form of the same transporter, thus making it fairly easy to generate and interpret data). It is important to realize that under more realistic *in vivo* conditions, the NBDs are exposed to ATP at millimolar or ADP at three‐digit micromolar concentrations [72] and hence fulfill their function being confronted with a sea of nucleotides that differ by one phosphate group.

The most 'radical' mechanism taking this reasoning into account was proposed for the bacterial lipid transporter PglK, which oscillates between outward‐occluded (Occ; ADP‐bound) and OF (ATP‐bound) states to flip lipid‐linked oligosaccharides across the membrane, while the IF apo state was assigned the insignificant role of a functionally irrelevant state [73]. In contrast, the lipid A transporter MsbA has been shown to bind its voluminous substrate in an IF cavity [74, 75]; without considerable NBD separation, the cavity would be insufficiently large to accommodate lipid A, and consequently, IF states of MsbA with separated NBDs must exist in the cell.

Further evidence for the existence of IF states under realistic conditions was provided in studies which investigated the conformational spectrum of ABC exporters under ATP hydrolysis conditions by double electron–electron resonance spectroscopy (DEER) (TM287/288, BmrCD, ABCB1), smFRET (ABCC1), or cryo‐EM (TmrAB) [15, 16, 61, 62, 71]. All these studies revealed that a considerable fraction of transporters adopted IF states with opened NBDs while hydrolyzing ATP. Hence, the debated tipi tent‐like conformations with opened NBDs likely also exist (at least for the majority of ABC exporters) in the presence of ATP inside metabolically active cells.

### Nucleotide‐bound structures at odds and in‐line with the prevailing alternating access model

In the continued discussion, we shall focus on type I ABC exporters, their remarkable functional diversity, and structural observations, which are at odds with the current mechanistic models.

The structural founding member of the type I ABC exporter class is Sav1866 [4]. The initial Sav1866 structure contained two molecules of ADP sandwiched at its fully closed NBDs, while the TMDs assume a widely opened OF conformation with separated wings at the extracellular gate [4]. Ironically, this first structure was at odds with the ATP‐switch model and numerous structural and biochemical studies of isolated NBDs, namely that only ATP is capable of inducing firm NBD closure; in the presence of ADP, the NBDs were expected to be separated and the TMDs to constitute an IF cavity. To establish harmony with the prevailing models, a Sav1866 structure with bound AMP‐PNP was published soon after, which did not differ from the ADP‐bound structure with exception of the presence of density for the γ‐phosphates of the nucleotide [76]. Unfortunately, neither an apo Sav1866 structure nor EPR studies on Sav1866 had been reported. Therefore, our knowledge regarding the energy landscape of this famous ABC exporter remains incomplete.

Soon after the Sav1866 structure, the widely discussed and questioned apo structures of MsbA were published, in which the NBDs are far apart and the TMDs assume a tipi tent‐like conformation with a large IF cavity [63]. While ADP‐bound structures of MsbA were not reported, the AMP‐PNP‐bound structure looked basically identical to OF Sav1866. Over the years, many more IF apo structures were reported. The majority of these structures confirmed the initial finding for MsbA, namely that in the absence of nucleotides, the NBDs are widely separated by variable degrees and do not contact each other [63, 64, 65, 73, 77, 78, 79, 80, 81]. There is also a growing number of cases, the first one being the apo structure of TM287/288, in which the NBDs maintain molecular contacts mediated by the D‐loop or C‐terminal extensions also in the apo state (Fig. 3A) [53, 82, 83, 84]. There are also several instances in which IF ABC exporters structures were determined in the presence of nucleotide analogues such as AMP‐PNP (e.g., TM287/288 or ABCB10) (Fig. 3B) [77, 85]. Hence, as opposed to the OF and occluded (Occ) structures of Sav1866 [76], MsbA [63], and McjD [86] obtained using AMP‐PNP, nonhydrolysable analogues proved to be insufficient to firmly close the NBDs and stabilize the OF or Occ state of these transporters. DEER analyses confirmed that nonhydrolyzable ATP analogues are frequently insufficient to close the NBD dimer (e.g. in case of TM287/288 [15], BmrCD [62]; TmrAB [87] and ABCB1 [16]), whereas for MsbA [69] it fully switches the transporter to the OF state. The inability of nonhydrolyzable ATP analogues to reliably stabilize the closed NBD dimer was frequently overcome by mutating the catalytic Walker B glutamate residue to glutamine, alanine, or glycine and thereby trap the Occ or OF state in the presence of ATP [52, 56, 59, 60, 61, 67, 88]. Cases in which the Walker B glutamate mutation trick did not work to obtain OF or outward‐occluded structures have not been reported.

It is finally interesting to note that despite all structures obtained in the presence of ATP, ADP, and ATP analogues (in particular the ones featuring unexpected conformations), the ATP‐switch model suggested in 2004 was never seriously challenged and still prevails [54].

### Substrate binding and transporter isomerization—the many unknowns of how this really works

ATPase activity of the great majority of ABC exporters is stimulated in the presence of substrates [53, 89, 90]. Although the degree of substrate‐stimulated ATPase activity is variable depending on the transporter, it is clear that the substrate‐binding site at the TMDs and the site of ATP hydrolysis at the NBDs are allosterically coupled. Unfortunately, many of the investigated ABC exporters transport hydrophobic compounds (in particular in case of multidrug efflux pumps), which complicates the biochemical and structural investigation of substrate binding. The first high‐quality structures with bound substrates were obtained for Atm1 of the organism *Novosphingobium aromaticivorans*, a type I ABC exporter involved in heavy metal detoxification [83]. Structures in complex of oxidized glutathione (GSSG) as well as with mercury glutathione (Hg‐GSSG) were reported (Fig. 3C). The substrates were found to be bound at the level of the inner membrane leaflet. Mutations of substrate‐binding residues resulted in a reduction of substrate‐stimulated ATPase activity, thereby validating the structural insights [83]. A crystal structure of MsbA obtained in complex with a strong inhibitor (G907) bound to a conserved region at the TMDs revealed a partial unlocking of coupling helices resulting in an asymmetric arrangement of the NBDs, which appears to be incompatible with NBD closure [75]. Cryo‐EM structures of MRP1 (ABCC1) as well as ABCB1 were reported in the apo and substrate‐bound states (Fig. 3C) [79, 80, 91]. For both transporters, substrate binding resulted in a contraction of the IF cavity. In case of ABCC1, leukotriene C_4_ interacts with both halves of the transporter and two pockets with opposing biophysical properties accommodate the amphiphilic substrate; the accommodated leukotriene C_4_ molecule is however still accessible for smaller molecules and water from the cytoplasm [80]. In case of ABCB1, one molecule of the substrate taxol or two molecules of the potent inhibitor zosuquidar were found to be occluded due to conformational changes at transmembrane helices (TMHs) 4 and 10, which form a cytoplasmic gate (inward‐occluded state) [91]. As a consequence of the contraction of the IF cavity concomitant with substrate binding, the NBDs of ABCB1 and ABCC1 approach each other [80, 91]. Nevertheless, strong molecular interactions between the NBDs are thereby not established. In contrast, the NBDs of Atm1 do not approach, which might be owing to the fact that these were crystal structures obtained by the same crystal form or that in case of Atm1 the NBDs remain in contact also in the absence of nucleotides *via* long C‐terminal helices [83]. At least for ABCB1 and ABCC1, cryo‐EM analysis suggests that substrate‐mediated reduction of NBD distance increases the chance of ATP‐mediated NBD closure. Intriguingly, the zosuquidar‐bound structure of ABCB1 also leads to a reduction of the inter‐NBD gap relative to the apo state. However, it places the two NBDs somewhat differently as compared to the taxol‐bound structure in a configuration which is probably inadequate for NBD closure [91], akin to the inhibitor‐bound MsbA structure [75]. Hence, the differences between substrate‐ and inhibitor‐mediated conformational changes with regard to NBD positioning are subtle, and on their own cannot satisfactorily explain stimulation or inhibition of ATPase activity.

Unfortunately, the IF‐OF isomerization cannot be followed by experimental approaches (e.g. smFRET), because it is too fast [71]. Further, there are seemingly no stable intermediates along the transition trajectory that would allow for structural analyses. This is why this important transition can only be investigated by molecular dynamics approaches. In recent unguided simulation studies, the heterodimeric ABC exporter TM287/288 was shown to isomerize from its IF to an OF conformation in response to ATP binding [17], and thereby to translocate substrates across the membrane [92]. Another unresolved question is whether and when the ATP molecules need to bind during NBD closure. Cross‐linking experiments on TM287/288 have shown that the closure also occurs in the absence of nucleotides [85]. Recent smFRET studies on ABCC1 and McjD have observed spontaneous NBD closure in the absence of substrates and ATP, which were however infrequent and short‐lived [10, 71]. These observations suggest that ATP binding is only needed toward the end of the trajectory, namely to firmly dimerize the NBDs and to initiate the subsequent hydrolysis reaction. Nevertheless, for many transporters the NBDs are always occupied with nucleotides (e.g. the IF structures of TmrAB under hydrolysis conditions [61] while in the case of TM287/288, the consensus ATP binding site exhibits distortions in the IF state that are incompatible with nucleotide binding [85].

## Potential roles of the extracellular gate in substrate release

Type I ABC exporter structures with fully dimerized NBDs bearing two sandwiched nucleotides are fixed points of the transport cycle, and have been obtained through various means (see above). The pertinent structures assume either the Occ or the OF state. While the structures of McjD [86] and Sav1866 [4] mark the fully closed and fully opened extracellular gates, respectively, other transporters (e.g. PglK, ABCB1, ABCC1) exhibit partial and/or asymmetric opening of the extracellular gate (Fig. 3D) [56, 59, 93]. For TmrAB, both the Occ and OF conformation were found to co‐exist according to cryo‐EM analyses [61]. Hence, it is at the extracellular gate where ATP‐bound ABC exporters with closed NBDs differ the most and are most flexible, a notion that is supported by DEER analyses on ABCB1, TM287/288, and TmrAB [15, 16, 87]. For classical ABC exporters, the Occ and/or the OF states (depending on the transporter) likely represent the conformations after substrate release. As a consequence, the exact sequence of events between the two structural fixed‐points, namely substrate‐bound IF‐conformation and substrate‐free Occ/OF conformation, is largely unknown and therefore remains speculative. The structural variations at the extracellular gate may reflect different mechanisms of substrate release; whereas for transporters with a high extracellular gate opening propensity, the substrate may be released *via* diffusion, a peristaltic mechanism could be at work to actively extrude the bound molecule in cases where the extracellular gate readily collapses. An interesting ‘outward‐only’ mechanism had been proposed for PglK [73]. According to this mechanism, lipid‐linked oligosaccharides are not accommodated in the IF cavity, but instead directly jump into an OF cavity that is stabilized by ATP binding at the NBDs. ATP hydrolysis is then required to eject the substrate, followed by extracellular gate closure to assume the ADP‐bound Occ state. Exchange of ADP by ATP then resets the transporter. While the unusual transport mechanism of PglK is mainly based on crystal structures and investigations of rationally introduced mutations *via* an elegant *in vitro* lipid flipping assay, further investigations with spectroscopy techniques would be very interesting to learn more about the role of the extracellular gate of this fascinating ABC exporter.

## Transporter resetting—the dark side of the cycle

While the IF‐OF isomerization was the focus of research for many years, the back‐transition to the IF state has only recently received more attention. It is generally accepted that ATP hydrolysis precedes NBD opening and dissociation [94]. In addition, recent studies of heterodimeric ABC exporters featuring both a degenerate and a canonical nucleotide‐binding site suggest that hydrolysis of one ATP molecule is sufficient to reset the transporter [57, 60, 61, 71]. A smFRET study on ABCC1 revealed that resetting is the rate‐limiting step of the transport cycle and that NBD dissociation rather than ATP hydrolysis represents the slow component [71]. A cryo‐EM investigation of TmrAB under turnover conditions revealed a large population of transporters assuming two states called ‘unlocked return’, which show slightly opened NBD‐bearing ADP at the consensus site and a still fully closed degenerate site with ATP bound, concomitant with a gradual opening of the intracellular gate (Fig. 3B) [61]. Hence, the full dissociation of the NBDs back to the IF state represents a strong kinetic barrier for TmrAB. In case of TM287/288, an OF‐state selective synthetic nanobody was used to follow the OF‐IF transition using SPR [60]. Using the Walker B E‐to‐Q mutant of TM287/288, it was shown that the OF state is very long‐lived and that OF‐IF isomerization is as slow as the residual ATPase activity of this mutant. This notion was also confirmed using ATP‐trapping assays with TmrAB [57]. Interestingly, two mutations weakening extracellular gate closure of TM287/288 resulted in a transporter with a markedly slower OF‐IF isomerization and a strongly decreased ATPase activity. Thus, firm closure of the extracellular gate is required to efficiently reset the transporter back to its IF state in addition to ATP hydrolysis [60]. The propensity of the extracellular gate to open and close, respectively, is therefore not only relevant in the context of uphill transport of substrates, but also profoundly shapes the energy landscape of ABC exporters.

## One instrument—many melodies

While the field closes in on a unified general mechanism of how the free energy of ATP is utilized to pump substrates across the membrane, there is a remarkable plurality of mechanistic details that exist among different type I ABC exporters. Besides the rather obvious differences of the physical–chemical properties of the large substrate‐binding pockets, which govern the substrate specificity or preference, there are two other elements that likely play an equally important role.

Firstly, structural peculiarities have been regularly uncovered and appear to play a profound functional role. For example, PglK was found to contain an additional extracellular helix connecting TMHs 1 and 2, which was shown to play an important role in lipid‐linked oligosaccharide transport *via* a non‐canonical ‘credit‐card swipe’ mechanism [73]. CFTR (ABCC7) has been dubbed a 'broken transporter' and was found to form an imperfect intracellular gate while the NBDs are dimerized, which ultimately allow this transporter to operate as a ATP‐gated chloride channel [88]. The mycobacterial type I ABC exporter Rv1819c contains an unusual extracellular cap formed by extended TMHs 1 and 2 and residues N‐terminal to an additional TMH 0. Together with a closed intracellular gate due to closed NBDs, an unusually large and hydrophilic cavity is formed, which is used to import vitamin B_12_ and other large hydrophilic compounds [52]. And finally, the mycobacterial siderophore transporter IrtAB contains a partially collapsed IF cavity due to several irregularities and breaks in its TMHs. This structural feature appears to be linked to IrtAB’s function, namely the import of iron‐charged siderophores into the cytoplasm [53].

A second underappreciated source for functional differences is the energy landscape. DEER and smFRET analyses have uncovered major differences among ABC exporters in terms of populating the various states of the transport cycle. This is observed by different reactions to ATP analogues (see above), but is also manifested under ATP hydrolysis conditions. In the presence of ATP‐Mg^2+^, ABCC1 and TM287/288 primarily adopt the OF state [15, 71], while BmrCD and ABCB1 primarily adopt IF states with various degrees of NBD opening [16, 62]. Cryo‐EM investigation of TmrAB under hydrolysis conditions could not detect OF or Occ states; instead the NBDs slightly open at the consensus site right after ATP hydrolysis [61]. Importantly, the energy landscape defines the dwell times a transporter rests in the thermodynamic minima of the cycle while it binds and hydrolyzes ATP and captures and ejects substrates. In how far the underlying kinetics influences substrate transport still awaits investigation.

## Concluding remarks

All biochemical and biophysical approaches are prone to potential artifacts and have their limitations. For example, smFRET, which is a very powerful technique to study conformational dynamics suffers from photoblinking and photobleaching, saturation effects, photoisomerization, and artificial high‐FRET states due to dye–dye interactions [95, 96, 97]. Such artifacts may lead to complications in data analysis and erroneous mechanistic conclusions. Similarly, EPR and DEER, another method frequently used to study the conformational states of ABC transporters, may suffer from artifacts attributed to high‐frequency noise and low modulations [98]. In addition, in order to perform smFRET or DEER analyses, the investigated transporter needs to be mutated to first remove all native cysteines and then to introduce cysteines at strategic positions to chemically attach the FRET and spin labels, respectively. All these modifications inevitably influence the functionality of the transporter and in some cases may lead to incorrect results.

In this opinion paper, we advocate that structural biology can be similarly afflicted by artifacts and complications in data interpretations. Therefore, structures should not be taken as gospel [99] and their mechanistic interpretation needs to be always conducted within the context of the available functional data and interrogated by orthogonal biochemical/biophysical approaches to reduce the risk of interpretation errors.

One major point to consider is the energy landscape of the transporter. Single‐molecule studies of ABC transporters demonstrated the mechanistic importance of conformational equilibrium and distribution, spontaneous conformational fluctuations, and conformational selection [11]. However, the energy landscape and conformational dynamics of any protein has been shaped by evolution to function and respond at physiological protein and ligand concentrations. In this respect, the high protein and ligand concentrations used for crystallization, and especially those found in the crystals themselves, likely have a Le‐Chatelier effect on the conformational dynamics, shifting the equilibrium to a distribution that is not necessarily physiologically relevant. This is especially relevant when trying to interpret the allosteric effects of ligands (nucleotides, SBP, substrate) on the conformational state, since a minor or an off‐trajectory conformation may have become overpopulated in the crystal lattice. A similar bias in conformational distribution may be caused by cross‐linking, covalently altering the energy landscape of the protein and shifting the conformational equilibrium.

Additional bias in interpretations of structures may arise from the presence of detergents. As discussed herein, the properties of ABC transporters are frequently found to differ between the detergent and membrane environments [20, 24, 35]. This is especially evident when examining allosteric effects, such as coupling between ATP hydrolysis and substrate/SBP binding, but also in the context of NBD separation and extracellular gate opening in ABC exporters. In this respect, the emerging use of cryo‐EM single particle analysis may provide an advantage in some cases as the structure can be determined in nanodiscs, which better mimic the membrane environment than detergents. Cryo‐EM single particle analysis also uses lower protein and ligand concentrations, potentially decreasing nonphysiological Le‐Chatelier effects.

Finally, to fully understand the transport mechanism and its energetics, the use of spectroscopic methods such as EPR, luminescence resonance energy transfer and single‐molecule FRET [11, 24, 36, 60, 100] and molecular simulations [9] will be insightful to assess (a) the dominant states of the transporter and the minor subpopulations or transient intermediates; (b) the amplitude and frequency of conformational motions; and (c) the sequence of the conformational changes and their connection to specific catalytic events such as ATP binding and hydrolysis [34]. Only *via* thorough investigations that employ these orthogonal methodologies, the increasing number of novel ABC transporter structures will come to live and the field advances its mechanistic understanding.
